# Investigating the role of small, acid-soluble spore proteins (SASPs) in the resistance of *Clostridium perfringens *spores to heat

**DOI:** 10.1186/1471-2180-6-50

**Published:** 2006-06-08

**Authors:** Deepa Raju, Michael Waters, Peter Setlow, Mahfuzur R Sarker

**Affiliations:** 1 Department of Biomedical Sciences, College of Veterinary Medicine, Oregon State University, Corvallis, Oregon 97331, USA; 2 Department of Microbiology, College of Science, Oregon State University, Corvallis, Oregon 97331, USA; 3 Department of Molecular, Microbial and Structural Biology, University of Connecticut Health Center, Farmington, CT 06030, USA

## Abstract

**Background:**

*Clostridium perfringens *type A food poisoning is caused by enterotoxigenic *C. perfringens *type A isolates that typically possess high spore heat-resistance. The molecular basis for *C. perfringens *spore heat-resistance remains unknown. In the current study, we investigated the role of small, acid-soluble spore proteins (SASPs) in heat-resistance of spores produced by *C. perfringens *food poisoning isolates.

**Results:**

Our current study demonstrated the presence of all three SASP-encoding genes (*ssp1*, *2 *and *3*) in five surveyed *C. perfringens *clinical food poisoning isolates. β-Glucuronidase assay showed that these *ssp *genes are expressed specifically during sporulation. Consistent with these expression results, our study also demonstrated the production of SASPs by *C. perfringens *food poisoning isolates. When the heat sensitivities of spores produced by a *ssp3 *knock-out mutant of a *C. perfringens *food poisoning isolate was compared with that of spores of the wild-type strain, spores of the *ssp3 *mutant were found to exhibit a lower decimal reduction value (*D *value) at 100°C than exhibited by the spores of wild-type strain. This effect was restored by complementing the *ssp3 *mutant with a recombinant plasmid carrying wild-type *ssp3*, suggesting that the observed differences in *D *values between spores of wild-type versus *ssp3 *mutant was due to the specific inactivation of *ssp3*. Furthermore, our DNA protection assay demonstrated that *C. perfringens *SASPs can protect DNA from DNase I digestion.

**Conclusion:**

The results from our current study provide evidences that SASPs produced by *C. perfringens *food poisoning isolates play a role in protecting their spores from heat-damage, which is highly significant and relevant from a food safety perspective. Further detailed studies on mechanism of action of SASPs from *C. perfringens *should help in understanding the mechanism of protection of *C. perfringens *spores from heat-damage.

## Background

*Clostridium perfringens *is a gram-positive, spore-forming, anaerobic bacterium that has long been recognized as a significant cause of histotoxic and gastrointestinal (GI) diseases in both humans and animals [1,2]. *C. perfringens *isolates can be classified into one of five types (A through E) based upon their ability to produce alpha-, beta-, epsilon- and iota-toxin [2]. Although enterotoxin (CPE)-producing *C. perfringens *type A isolates represent <5% of global *C. perfringens *population, these bacteria are very important human GI pathogens, causing *C. perfringens *type A food poisoning and non-food-borne GI diseases [1]. Substantial experimental and epidemiological evidence [1,3] now indicates that CPE plays a major role in the development of GI symptoms in cases of *C. perfringens*-associated food poisoning and non-food-borne GI diseases. In addition to producing CPE, *C. perfringens *food poisoning isolates have the ability to form heat-resistant spores, which can survive boiling for an hour or longer [1,4,5]. The possession of high spore heat-resistance facilitates the survival of *C. perfringens *spores in primary food vehicles (such as meat and poultry products) where, in the presence of appropriate nutrients, dormant spores are reverted to vegetative cells and multiply, and then cause food-borne illness after consumption of these contaminated foods [1].

The molecular basis for *C. perfringens *spore heat-resistance remains unknown. However, since small, acid-soluble spore proteins (SASPs) from *Bacillus subtilis *can protect their spores from damage by heat and other environmental factors [6-8], it is very likely that *C. perfringens *SASPs play a similar role. Spores of *Bacillus *and *Clostridium *species contain a number of SASPs of molecular weight 5–7 kDa, which comprise 10–20% of the total spore protein [8]. These proteins are classified into two groups based on their primary sequence, an α/β-type and a ϒ-type [8]. The *B. subtilis *α/β-type SASPs are encoded by multiple genes and comprise a large protein family whose amino acid sequences are very highly conserved within and between species [8]. The *B. subtilis *α/β-type SASPs are non-specific DNA binding proteins which saturate the spore chromosome and protect spore DNA from damage caused by heat, UV radiation, and peroxidase [9-12]. Although an enormous number of studies on the role of SASPs in *B. subtilis *spore resistance have been carried out, nothing is known about the role of *C. perfringens *SASPs. However, the advance towards understanding the role of SASPs in *C. perfringens *spore resistance is emerging from some old studies [13-15] which revealed the existence of multiple α/β-type, but not ϒ-type, SASPs in *Clostridium *species. Three genes (*ssp1*, *2 *and *3*) coding for α/β-type SASPs have been cloned and nucleotide sequenced from *C. perfringens *old strains [16,17]. Interestingly, only these three *ssp *genes, compared to at-least 7 *ssp *genes in *B. subtilis *[8], were identified in the genome of *C. perfringens *strain 13 [18]. However, the presence and expression of these *ssp *genes in recent clinical *C. perfringens *food poisoning isolates and their role in spore heat-resistance remains unknown.

In the current study, we investigated the genetics and expression of *ssp *genes in *C. perfringens *clinical food poisoning isolates. Our study also prepared an isogenic *ssp3 *knock-out mutant of a *C. perfringens *type A food poisoning isolate. The heat sensitivities of spores produced by *ssp3 *knock-out mutant was compared with that of spores of the wild type strain. The results from these experiments now provide evidence that *C perfringens *SASPs play a role in spore heat-resistance.

## Results

### PCR screening and comparison of *ssp *ORFs in *C. perfringens *food poisoning isolates

**Figure 1:**
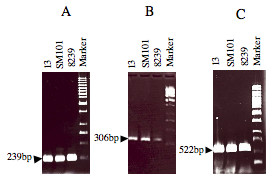
*ssp *PCR analysis of *C. perfringens *food poisoning isolates. Representative results of a PCR assay with primers designed to amplify *ssp1 *(A), *ssp2 *(B) or *ssp3 *(C) specific PCR products. Results are shown for control strain 13 (a known *ssp1*, *2 *and *3*-positive strain) and representative food poisoning isolates SM101 and NCTC8239. Marker indicates the migration of DNA size markers (Gene Ruler™ 1-kb ladder; Fermentas).

We first subjected our collection of *C. perfringens *food poisoning isolates to *ssp*-specific PCR analysis to determine whether most, or all, *cpe*-positive food poisoning isolates carry all three *ssp *genes (*ssp1*, *2 *and *3*). Control PCRs were run using template DNA prepared from a known *ssp*-positive *C. perfringens *strain 13 [18]. When template DNA isolated from each of our five surveyed clinical *C. perfringens *food poisoning isolates was subjected to this same *ssp *PCR analysis, PCR products of 239-, 306-, and 522-bp were invariably obtained (see Fig. 1 for representative results). These results are in consistent with the known presence (Fig. 1) of all three *ssp *ORFs in the genome of the *C. perfringens *strain 13 [18].

The nucleotide sequencing analyzes revealed (data not shown) that no mutations or termination codons were found in the *ssp1*, *2 *or *3 *ORF sequences of our five surveyed isolates that encode proteins of 60-, 59- or 60-aa, respectively. The *ssp1*, *2 *or *3 *ORF sequences and also the upstream promoter sequences are highly conserved in all our surveyed food poisoning isolates and matches exactly with previously published sequences [16,18].

### Evaluation of expression of *ssp *genes in *C. perfringens *food poisoning isolates

To examine the expression of *ssp *genes, the putative promoter regions of *ssp1*, *2*, or *3 *from *C. perfringens *food poisoning isolate SM101 was fused to *E. coli gusA*. These *ssp-gusA *fusion constructs were introduced into wild-type SM101 and β-glucuronidase (GUS) activity was measured during vegetative and sporulation growth. A promoter-less *gusA *construct was used as a negative control to ensure the specificity and reliability of the assay (Data not shown).

**Figure 2:**
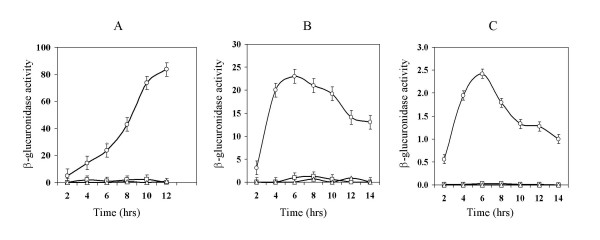
Expression of *ssp1-gusA *(A), *ssp2-gusA *(B) and *ssp3-gusA *(C) fusions in *C. perfringens *wild-type SM101 grown in vegetative (□) and sporulation (○) medium, and in *spo0A *mutant IH101 grown in sporulation medium (Δ). β-glucuronidase activity (in Miller Units) was calculated as previously described [26, 28]. Data represents the average of three independent experiments.

When GUS assay was performed on SM101 carrying pSG12 (*ssp1*-*gusA*), pSG22 (*ssp2-gusA*) or pSG32 (*ssp3*-*gusA*), no significant expression of *ssp1*, *2 *or *3 *was observed in vegetative cells (Fig. 2). However, SM101 carrying *ssp1-*, *2*-or *3*-*gusA *showed GUS activity during sporulation. The *ssp1*-*gusA *expression began at ~4 h after induction of sporulation, with maximum specific activity attained at ~12 h after induction of sporulation (Fig. 2). Both *ssp2-gusA *and *ssp3-gusA *fusions were expressed beginning at ~2 h after induction of sporulation, with maximum specific activity attained ~6 h after induction of sporulation (Fig. 2). Collectively, these results suggest that all three *ssp *genes are expressed during sporulation, but not during vegetative growth.

To analyze the sporulation-dependent expression of *ssp *genes further, we examined the expression of *ssp1*-, *2*- or *3*-*gusA *fusion in a *spo0A *mutant of *C. perfringens*. The rationale for using *spo0A *mutant is that, if *ssp *expression is truly dependent on sporulation, *ssp-gusA *fusions would not be expressed in sporulation-deficient *spo0A *mutant of *C. perfringens *[19]. The *ssp*-*gusA *fusion constructs pSG12 (*ssp1*-*gusA*), pSG22 (*ssp2-gusA*) and pSG32 (*ssp3*-*gusA*) were introduced into *C. perfringens spo0A *mutant strain IH101 and GUS activity was measured during sporulation growth. As shown in Fig 2, no detectable GUS activity was obtained with sporulating cultures of IH101 carrying *ssp1*-, *2*, and *3*-*gusA *fusions, confirming that the expression of *ssp *genes in *C. perfringens *are dependent on *spo0A *expression and sporulation.

### SASP production by *C. perfringens *food poisoning isolates

**Figure 3:**
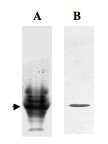
Production of SASPs by *cpe*-positive *C. perringens*. (A) SASPs, prepared from a representative *C. perfringens *food poisoning isolate SM101, was separated by polyacrylamide gel electrophoresis at low pH and stained with Coomassie brilliant blue. (B) Western blot of the gel shown in panel A. The blot was probed with antibodies against *B. subtilis *SspC [13] and developed by chemiluminescence detection to identify immunoreactive species. Arrow indicates the major band that was immunoreactive with *B. subtilis *SspC antibody.

Having obtained evidence that all three *ssp *genes are expressed in *C. perfringens *food poisoning isolates, we next examined whether these isolates can, in fact, produce SASPs. When acid-extracts of spores from representative *C. perfringens *food poisoning isolates SM101 (Fig. 3) and NCTC8239 (data not shown) were analyzed by polyacrylamide gel electrophoresis at low pH, multiple protein bands were observed. Western blot analysis, using antibodies against SspC of *B. subtilis*, detected SspC-specific immunoreactivity in acid-extracts of spores from SM101 and NCTC8239 (Fig. 3 and data not shown). These results indicated that *C. perfringens *food poisoning isolates can produce SASPs.

### Creation of *ssp *knock-out mutants

To evaluate the role of SASPs in spore heat-resistance, we attempted to introduce knock-out mutations into each *ssp *gene. The mutator plasmid pDR63, pDR27 or pMRS62, carrying mutated allele Δ*ssp1*::*catP*, Δ*ssp2*::*catP *and Δ*ssp3*::*catP*, respectively, was introduced into *C. perfringens *strain SM101 by electroporation, and Em^r^Cm^r ^transformants were selected. As a positive control of transformation, the *C. perfringens*-*E. coli *shuttle plasmid pJIR751 encoding Em^r ^was included. The Em^r^Cm^r ^transformants were obtained at ~1000-fold less frequencies than that of pJIR751-derived Em^r ^transformants, which was expected because mutator plasmids pDR63, pDR27 and pMRS62 have no origin of replication for *C. perfringens*. The single cross-over event of homologous recombination was confirmed by PCR using each *ssp-*specific primers (data not shown). One transformant for each mutator plasmid was grown in the absence of both Em and Cm, and a double crossover event between each wild-type *ssp *and the respective mutated Δ*ssp*::*catP *allele was screened by selecting colonies for Cm^r ^and Em^s ^phenotypes. After screening of ~2,500 colonies from SM101(pMRS62) culture, we obtained one putative clone showing a Cm^r ^and Em^s ^phenotype. This putative *ssp3 *mutant was designated as DR101. However, no Cm^r ^and Em^s ^clone was obtained after screening of >3,000 colonies from each of SM101(pDR27) and SM101(pDR63) culture. Our three repeated attempts using SM101(pDR63) and SM101(pDR27) transformants from three independent electroporations were failed to isolate *spp1 *and *ssp2 *mutant, respectively.

### Confirmation of DR101 as *ssp3 *knock-out mutant

**Figure 4:**
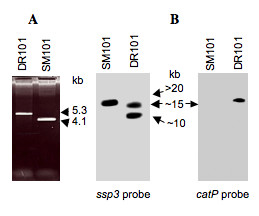
Molecular analysis of *ssp3 *knock-out mutant. (A) PCR analysis of the total DNAs from *C. perfringens *wild-type and *ssp3 *mutant. Total DNA isolated from wild type SM101 and the *ssp3 *mutant DR101 strains was subjected to PCR analysis using *ssp3*-specific primer pair CPP13/CPP16. (B) Southern hybridization analysis of total DNAs from *C. perfringens *wild-type and *ssp3 *mutant. Two identical Southern blots were prepared using *Hpa*I-digested DNAs from wild-type (SM101) and *ssp3 *mutant (DR101) and probed separately with either *ssp*-specific or *catP*-specific DIG-labeled probes. The migration of the hybridizing band derived from each strain is indicated between the two blots.

Inactivation of *ssp3 *in DR101 was first demonstrated by PCR analysis (Fig. 4A) using *ssp3*-specific primers. An ~4.1-kb fragment carrying *ssp3 *was amplified from DNA of wild-type strain SM101. In contrast, an ~5.4-kb PCR product was obtained from DNA of mutant strain DR101. These PCR results are consistent with the wild-type *ssp3 *gene in DR101 having been replaced with the mutated allele, carrying an extra ~1.3-kb *catP*-containing fragment.

Southern blot analyses showed that a single *Hpa*I-digested DNA fragment from wild-type strain SM101 hybridized with *ssp3*-specific probe (Fig. 4B). However, two hybridizing bands were observed with DNA from mutant strain, DR101 (Fig. 4B). This profile is consistent with results expected since the ~1.3-kb *catP*-containing fragment has an internal *Hpa*I site. The *catP*-specific probe hybridized with an ~15-kb *Hpa*I fragment of DR101 DNA, but as expected, no hybridizing band was observed with DNA from wild type SM101 (Fig. 4B). These results further confirmed that wild-type *ssp3 *gene in DR101 having been replaced with Δ*ssp3*::*catP *allele.

### Sporulation and CPE production by *ssp3 *mutant

We first compared the sporulation capability of the *ssp3 *mutant against that of its wild-type. Both the mutant strain DR101 and wild-type strain SM101 exhibited significant sporulation, i.e., refractile endospores were visualized by phase-contrast microscopy after 8 h growth in DS medium (data not shown). When we compared the CPE producing capabilities of the wild-type strain SM101 and *ssp3 *knock-out mutant DR101, an ~35-kDa CPE-specific immunoreactive band was detected in Western blots of lysates prepared from sporulating cultures of both SM101 and DR101 (data not shown). Quantitative analyses demonstrated similar level of CPE production in both SM101 and DR101 (data not shown).

### Comparison of the heat sensitivities of spores produced by *C. perfringens *food poisoning isolate carrying wild-type versus knock-out *ssp3*

**Figure 5:**
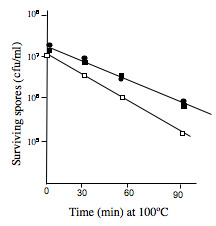
Thermal death curves for sporulating cultures of wild-type SM101 (●), *ssp3 *mutant DR101 (□), and complemented DR101(pDR18) (■) strains. The data is the result of a representative experiment; these results were highly reproducible (data not shown).

In order to determine whether *ssp3 *has any role in spore heat-resistance, we performed experiments to evaluate the heat sensitivities of spores produced by *C. perfringens *wild-type and *ssp3 *mutant. Representative thermal death curves obtained at 100°C for heat-shocked sporulating cultures of wild-type SM101 and *ssp3 *mutant DR101 are shown in Fig. 5. From the Fig. 5 results, we calculated that spores of SM101, carrying the wild-type *ssp3 *gene, had a *D *value of ~90 min at 100°C, while spores of DR101, an isogenic *ssp3 *mutant, had a *D *value of ~60 min. To further confirm our results, three additional *D *values at 100°C for heat-shocked sporulating cultures of DR101 and SM101 were determined using three independent thermal death curves (data not shown). These results demonstrated that spores produced by the *ssp3 *mutant had, on average, approximately 0.5-fold-lower *D *value at 100°C than spores produced by the wild-type strain (the difference was statistically significant at *P *= 0.001).

In order to determine whether the observed differences in *D *values between spores of SM101 and spores of DR101 was due to the specific inactivation of *ssp3*, *D *value was determined at 100°C for heat-shocked sporulating cultures of complemented strain DR101(pDR18). As calculated from Fig. 5, spores produced by DR101(pDR18) had a wild-type level *D *value i.e., ~90 min at 100°C. This finding was confirmed by three independent experiments. Collectively, our results indicated that the reduced heat-resistance of spores produced by the *ssp3 *knock-out mutant was caused by the specific inactivation of *ssp3*.

### Protection of plasmid DNA against DNase I

**Figure 6:**
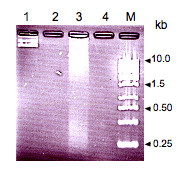
Protection of DNA against DNase I digestion by SASPs binding. The pJIR751 DNA was incubated alone (lane 1), with DNase I (lane 2), with SASPs and DNase I (lane 3) or DNase I in the presence of 8 M urea (lane 4). M indicates the migration of DNA size markers (Gene Ruler™ 1-kb ladder; Fermentas). The results are from representative experiments; these results were highly reproducible (data not shown).

Since *B. subtilis *SASPs have been shown to bind double-stranded DNA and protect against DNase cleavage [20], we wondered whether *C. perfringens *SASPs can do the same. We purified total SASPs from *C. perfringens *SM101 (Fig. 3) and performed SASP-DNA binding assay by measuring the ability of SASPs to protect plasmid (pJIR751) DNA from DNase I digestion. As shown in Fig. 6., when pJIR751 DNA was incubated with DNase I, no DNA band was observed after agarose gel elctrophoresis (lanes 2 and 4), indicating that DNase I is enzymatically active and completely digested the pJIR751 DNA. However, when pJIR751 DNA was incubated with *C. perfringens *SASPs prior to DNase I treatment, discrete DNA fragments remained after DNase I treatment (lane 3). Furthermore, the incomplete digestion of SASP-treated DNA was not due to the inhibition of DNase I activity by urea because the enzyme was completely functional in the presence of 8 M urea (Fig. 6, lane 4) that was used to dissolve SASPs. Collectively, these results indicated that SASPs protected plasmid DNA against DNase I digestion. Similar results were obtained in our three independent experiments (see Fig. 6 for representative results). This observation indicated that the function of SASPs in *C. perfringens *food poisoning isolates is similar to that in *B. subtilis*, i.e., they protect spores from heat-damage by binding to spore DNA.

## Discussion

*C. perfringens *type A food poisoning, which currently ranks as the third most commonly reported food-borne disease in the United States [1], is caused by enterotoxigenic *C. perfringens *type A isolates that typically possess high spore heat-resistance [1,4]. The possession of high spore heat-resistance favors the survival of *C. perfringens *food poisoning isolates during incomplete cooking or inadequate holding of foods, which are the two major factors contributing to *C. perfringens *type A food-borne illness [1]. However, the molecular basis for *C. perfringens *spore heat-resistance remains unknown. In the current study, we hypothesized that SASPs produced by *C. perfringens *food poisoning isolates can play a role in the resistance of their spores to heat.

Our current study demonstrated the presence of all three *ssp *genes in a large number of clinical *C. perfringens *food poisoning isolates. Nucleotide sequencing revealed that the *ssp *ORFs in our surveyed isolates are intact, i.e., no mutations or premature termination codons were detected in the ORFs. Although previous studies [16,17] reported the presence of *ssp1*, *2 *and *3 *in *C. perfringens *laboratory strains, to our knowledge, our study is the first to compare all three *ssp *ORFs between a large number of clinical *cpe*-positive *C. perfringens *food poisoning isolates. The deduced amino acid sequence of SASPs from our surveyed *C. perfringens *food poisoning isolates are identical to that of the *C. perfringens *SASPs published earlier [16,17] and homologous to α/β-type SASPs of *B. subtilis *[8] indicating that the *ssp *ORFs present in food poisoning isolates are indeed *ssp *genes.

This study report evidences that the *ssp *genes present in *C. perfringens *food poisoning isolates are functional. Our GUS assay showed that *ssp1*, *2 *and *3 *from a food poisoning isolate SM101 were expressed during sporulation, but not during vegetative growth, and expression begins ~3–4 h after induction of sporulation. These results suggest that the mechanism of regulation of *C*. *perfringens *SASPs may be similar as that of *B. subtilis*. In *B. subtilis*, SASPs synthesis begins 3–4 h into sporulation, when all three major SASPs are synthesized in parallel [8]. The differential expression of *ssp *genes (Fig. 2) can not be explained by the differences in ribosome binding sites (rbs) because the putative rbs is highly conserved among the three *ssp *genes (data not shown). Further support for the sporulation-dependent expression of *C. perfringens ssp *genes came from our observations that no GUS activity was detected in *spo0A *mutant IH101 carrying *ssp1*-, *2*- or *3*-*gusA *fusion. Consistent with these expression results, our study also provides evidence that *C. perfringens *food poisoning isolates can, in fact, produce SASPs. The multiple protein bands obtained in our study from acid extracts of spores produced by food poisoning isolates (Fig. 3) were also observed previously [13] in acid extracts of spores produced by *C. perfringens *NCTC9268. The identity of these acid-soluble spore proteins as SASPs was confirmed by Western blot analyses using *B. subtilis *SspC antibody (Fig. 3). The single immunoreactive band observed in Western blot can be explained by the fact that *C. perfringens ssp1*, *2 *and *3 *are highly homologous (>90%) to each other and encode similar sized proteins (59–60 aa). Collectively, the presence and expression of *ssp *genes in *C. perfringens *food poisoning isolates, which possess high spore heat-resistance as indicated in a previous study [4], significantly strengthen the hypothesis that SASPs are associated with heat-resistance of spores produced by these isolates.

The present study's most significant finding is the presentation of the first genetic evidence that *C. perfringens *SASPs play a role in heat-resistance of spores produced by *C. perfringens*. The inactivation of *ssp3 *significantly affected the heat-resistance of spores produced by a food poisoning isolate SM101. Our findings that i) spores of *ssp3 *knock-out mutant exhibited lower heat-resistance than that of the spores of wild type and ii) reversion of this effect by complementing the mutant with a recombinant plasmid carrying wild type *ssp3*, provided direct genetic evidence supporting the strong linkage between the production of SASPs and the resistance of spores to heat. The slight reduction of *D *value in spores of *ssp3 *mutant compared to the *D *value of spores of wild-type can be explained by the presence of the functional *ssp1 *and *ssp2 *genes in *ssp3 *mutant strain DR101. Our DNA protection assay also supports the role of *C. perfringens *SASPs in spore heat-resistance by demonstrating that *C. perfringens *SASPs, like *B. subtilis *SASPs [20], can protect plasmid DNA from DNase I digestion. Further studies on characterization of SASP-DNA binding and the effect of this binding on plasmid topology should help in understanding the mechanism of interaction between DNA and SASPs from *C. perfringens*.

To our knowledge, this report represents the first successful study involving the construction of a *C. perfringens ssp *knock-out mutant. The greatest challenge faced in our *ssp *knock-out study was the lack of an easy screening method for the second cross-over event. Although in our first attempt, using the double-antibiotic selection strategy [3], we were able to isolate *ssp3 *knock-out mutant, our three similar independent attempts were failed to isolate *ssp1 *and *ssp2 *mutant. The reasons for these failures can not be explained by the lack of sufficient amount of homologous DNA in mutator plasmids [21] because, both pDR62 and pDR27 carry at-least 1.4-kb homologous DNA located on either side of the insertionally inactivated *ssp *gene that should be sufficient to allow double-reciprocal crossover event [19]. It is also unlikely that incorporation of *catP *in *ssp1 *or *ssp2 *can cause polar effect on the downstream gene whose expression is essential for the survival of *C. perfringens *cells because our nucleotide sequencing analyses (data not shown) demonstrated that neither *ssp1 *nor *ssp2 *forms an operon with any downstream gene in the genome of SM101. Therefore, further research is needed to identify the relevant obstacles for isolating *ssp1 *and *ssp2 *mutants.

## Conclusion

The current study demonstrated that i) all three *ssp *genes are present and expressed in a large number of clinical *C. perfringens *food poisoning isolates, ii) *ssp3 *knock-out mutant of *C. perfringens *food poisoning isolate possess lower spore-heat resistance compared to that of its parent strain and this effect could be restored by complementing the mutant with wild-type *ssp3 *gene, and iii) SASPs from *C. perfringens *food poisoning isolate can protect DNA from DNase I digestion. Collectively, these results support our initial hypothesis that SASPs produced by *C. perfringens *food poisoning isolates can play a role in resistance of their spores to heat. These findings are highly significant and relevant from a food safety perspective because the possession of high spore heat-resistance favors the survival of *C. perfringens *food poisoning isolates in primary food vehicles (such as, meat and poultry products) contributing to *C. perfringens *food-borne illnesses [1]. Further detailed studies on mechanism of action of SASPs from *C. perfringens *should help in understanding the mechanism of protection of *C. perfringens *spores from damage caused by heat and other environmental stresses.

## Methods

### Bacterial strains and growth conditions

*C. perfringens *strains and plasmids used in this study are listed and described in Table 1. Starter culture (10 ml) of each *C. perfringens *isolate was prepared by overnight growth at 37°C in fluid thioglycollate broth (FTG) (Difco) as described previously [22]. For DNA isolation, an aliquot (0.2 ml) of each FTG culture was inoculated into 10 ml of TGY broth (3% Trypticase, 2% glucose, 1% yeast extract, 0.1% cysteine [22]) which was then incubated at 37°C for 18 h without shaking. For selecting *C. perfringens *transformants carrying recombinant plasmids, *C. perfringens *cultures were plated on Brain Heart Infusion agar plate containing erythromycin (50 μg/ml) or chloramphenicol (20 μg/ml) and incubated at 37°C. Sporulating cultures of *C. perfringens *were prepared by inoculating 0.2 ml of starter FTG medium culture into 10 ml of Duncan-Strong (DS) sporulating medium [22], which was then incubated for 24 h at 37°C. The presence of sporulating cells in each DS medium culture was confirmed by phase-contrast microscopy [19,23].

### *ssp*-specific PCR analysis

Total *C. perfringens *DNA was isolated from the overnight TGY medium cultures using a previously described protocol [4,24]. The isolated DNA was then subjected to *ssp *PCR analysis using primers specific for each of the *ssp1*, *2 *or *3 *genes as designed based on *C. perfringens *strain 13 genome sequence [18] (Table 2). Although the SASPs are multigene family proteins and the coding regions of three *C. perfringens ssp *are very similar, no sequence similarities were found between the flanking regions of these genes [16,18]. Therefore, each PCR primer pairs (Table 2) designed from the flanking region of each of the *ssp *gene amplified the respective *ssp *ORF sequences. These PCRs utilized 100 ng of template DNA, 25 pM of each primer, 200 μM deoxynucleoside triphosphates (dNTPs) (Roche), 2.5 mM MgCl_2_, and 1 U of *Taq *DNA polymerase (Fermentas) in a total volume of 50 μl. The reaction mixture was placed in a thermal cycler (Techne) for an initial period of 5 min at 94°C, then 28 cycles, each consisting of 1 min at 94°C, 1 min at 43°C (for CPP7/CPP8 and CPP9/CPP10) or 44°C (for CPP11/CPP34), 1 min at 72°C, and followed by an additional period of extension for 10 min at 72°C. After PCR, the presence of an amplified product was analyzed by subjecting an aliquot of each PCR sample to agarose (1.5%) gel electrophoresis.

### Cloning and sequencing of the *ssp*-containing fragments from various *cpe*-positive isolates

The DNA fragment containing *ssp *ORFs and an ~200 bp upstream sequence from each of five *C. perfringens *chromosomal *cpe *isolates was amplified by PCR as described above. These PCR products were then cloned into pCR^®^-XL-TOPO^® ^vector using the TOPO^® ^XL cloning kit (Invitrogen). Both strands of the *ssp-*containing DNA insert present in recombinant pCR^®^-XL-TOPO^® ^plasmid was then sequenced using M13 forward and reverse primers.

### Construction of *gusA*-fusion plasmids and β-glucuronidase assay

The *gusA *reporter vector pSM242 (obtained from Dr. Melville as a gift), is a derivative of an *E. coli-C. perfringens *shuttle vector pJIR750 [25], encoding chloramphenicol resistance (Cm^r^), contains the following features: i) four tandem terminators to minimize vector-based transcription, ii) multicloning sites located upstream of a promoterless *cpe*-*gusA *fusion, iii) the ribosome binding site and the first 13 amino acids of the *cpe *gene coding region to provide efficient translation [26] and iv) the *E. coli gusA *[27] as a transcriptional reporter element. Since our *C. perfringens spo0A *mutant already contained Cm^r ^marker, for our study we first constructed an erythromycin resistant (Em^r^) derivative of pSM242. An ~2.5-kb *EcoR*I-*Hin*dIII fragment of pSM242 was cloned into *Eco*RI/*Hin*dIII sites of an *E. coli-C. perfringens *shuttle vector pJIR751 [25], which encodes Em^r^, to create pMRS127.

The PCR amplified product (~200-bp) carrying the upstream region of each *ssp *was first cloned into pCR^®^-XL-TOPO^® ^vector using TOPO^®^-XL cloning kit (Invitrogen). Briefly, the DNA fragment carrying the promoter region of each of *ssp1*, *2 *or *3 *from SM101 (a food poisoning isolate carrying *cpe *on the chromosome) was amplified by PCR using primers CPP63/CPP64, CPP65/CPP66 and CPP57/CPP58, respectively (Table 2). The *Sal*I site was incorporated in the forward and *Pst*I site in the reverse primers of each primer pairs. These PCR products were then cloned into pCR^®^-XL-TOPO^® ^vector. The *Sal*I-*Pst*I fragments carrying the promoter regions of *ssp1*, *2 *or *3 *from pCR^®^-XL-TOPO^® ^clones were then re-cloned into the *Sal*I/*Pst*I sites of pMRS127 to create *ssp1-*, *2*- or *3-gusA *fusion constructs, pSG12, pSG22 or pSG32, respectively. As a negative control, pMRS130 carrying promoter-less *gusA *was constructed by cloning of *Xba*I-*Sac*I fragment from pSM104 [26] into the *Xba*I/*Sac*I sites of pJIR751. These plasmids were then introduced into *C. perfringens *wild-type SM101 or *spo0A *mutant IH101 by electroporation [24] and Em^r ^transformants were selected. The SM101 and IH101 transformants carrying *ssp-gusA *fusions or promoter-less *gusA *were grown in vegetative and sporulation conditions and tested for β-glucuronidase (GUS) activity as previously described [26,28].

### Extraction of SASPs and Western blotting

*C. perfringens *SASP was extracted using the protocol as previously described [29]. Briefly, sporulating cells of *C. perfringens *were sonicated and then centrifuged. The spores were washed several times with distilled water and lyophilized. The dried spores were subjected to dry rupture in a dental amalgamator (Wig-L-Bug) using 0.1 g of glass beads as an abrasive. The spore powder was resuspended in 3% acetic acid solution and then subjected to dialysis (Spectr/por^®^3, MWCO- 3,500, Spectrum laboratories) against 1% acetic acid solution at 4°C for at least 24 h. The sample was lyophilized and used for analysis by polyacrylamide gel electrophoresis at low pH as previously described [29]. The gel was run at 20 mA with the appropriate electrode polarity since SASPs are positively charged and hence run towards the cathode. The gel was stained with commassie brilliant blue (Bio-Rad) or transferred to a nitrocellulose membrane for Western blotting. The blot was probed with antibodies against *B. subtilis *SspC [13,29] and developed for chemiluminescence detection (Pierce) to identify immunoreactive species.

### Construction of mutator plasmids for *ssp1*, *2 *and *3*

*ssp1 *mutator plasmid: A 3306-bp DNA fragment containing *ssp1 *ORF and ~1.5-kb each upstream and downstream region was PCR amplified from genomic DNA of SM101 using primers CPP45 and CPP139 (Table 2) and cloned into pCR^®^-XL-TOPO^® ^(Invitrogen) to create pDR31. The 15-bp (nucleotides 80–94 from ATG) internal deletion as well as a unique *Nru*I site was incorporated in *ssp1 *in pDR31 by site-directed mutagenesis using Quick Change Mutagenesis System (Qiagen) using primers CPP104 and CPP105 to create pDR61. Plasmid pDR62 was construced by cloning a 1.3-kb *Sma*I*-Nae*I fragment of pJIR418, carrying chloramphenicol resistance marker *catP*, into *Nru*I site of pDR61. The *ssp1 *mutator plasmid, pDR63, was then constructed by re-cloning the *BamH*I-*Xho*I fragment containing Δ*ssp1*::*catP *allele, from pDR62 into *Bam*HI/*Sal*I sites of suicidal plasmid pMRS104.

*ssp2 *mutator plasmid: A 3081-bp DNA fragment containing *ssp2 *ORF and ~1.4-kb each upstream and downstream region was PCR amplified from genomic DNA of SM101 using primers CPP37 and CPP38 (Table 2) and cloned into pCR^®^-XL-TOPO^® ^(Invitrogen) to create pDR13. Plasmid pDR14 was constructed by deleting 15-bp (nucleotides 102–115 from ATG) internal *ssp2 *fragment as well as incorporating a unique *Nru*I site in *ssp2 *present in pDR13 by site-directed mutagenesis using Quick Change Mutagenesis System (Qiagen) using primers CPP47 and CPP48. A 1.3-kb *Sma*I*-Nae*I fragment of pJIR418, carrying chloramphenicol resistance marker *catP*, was cloned into *Nru*I site of pDR14 to create plasmid pDR26. The *ssp2 *mutator plasmid pDR27 was then constructed by re-cloning the *Bam*HI-*Xho*I fragment containing Δ*ssp2*::*catP *allele, from pDR62 into *Bam*HI/*Sal*I sites of suicidal plasmid pMRS104.

*ssp3 *mutator plasmid: A 4129-bp DNA fragment, carrying the *ssp3 *ORF and ~1.9-kb each upstream and downstream region, was PCR amplified from genomic DNA of SM101 using primers CPP13 and CPP16 (Table 2). This PCR fragment was cloned into pCR^®^-XL-TOPO^® ^(Invitrogen) to create the plasmid pBH2. A unique *Hpa*I restriction site was incorporated into the *ssp3 *ORF (at position 117 from ATG) present in pBH2 by site-directed mutagenesis using Quick Change Mutagenesis System (Qiagen) using primers CPP21 and CPP22 to create pMRS60. The *catP *gene was then inserted into the unique *Hpa*I site located within the *ssp3 *ORF in pMRS60 by digesting pMRS60 with *Hpa*I and ligating with a 1.3-kb *Sma*I-*Nae*I fragment containing the *catP *gene from pJIR418, to create pMRS61. The *ssp3 *mutator plasmid pMRS62 was then constructed by re-cloning the *Kpn*I-*Xho*I fragment of pMRS61 into *Kpn*I/*Sal*I sites of suicidal plasmid pMRS104.

### Isolation of *ssp *knock-out mutants

The mutator plasmids pDR63, pDR27 and pMRS62 were used to transform, by electroporation [24], *C. perfringens *isolate SM101 to Erythromycin (Em) (50 μg/ml) and Chloramphenicol (Cm) (20 μg/ml) resistance. One transformant for each plasmid was grown in TGY broth without any antibiotics and colonies sensitive to Em, but resistant to Cm were selected as previously described [3,19].

### Southern blot analysis

The *ssp3*-specific DNA probe was prepared using a 4129-bp DNA fragment, carrying *ssp3 *and ~1.9-kb each upstream and downstream region. This DNA fragment was amplified by PCR from genomic DNA of SM101 using primers CPP13 and CPP16 (Table 2). The *catP *probe was produced using a 517-bp *Eco*RV-*Hpa*I fragment, containing internal *catP *gene sequences, from pJIR418. These *ssp3*- and *catP*-containing DNA fragments were labeled using a Random Primed DNA Labeling system (Roche). Total DNA from wild type and *ssp3 *mutant strains was digested with *Hpa*I and two identical Southern blots were prepared using this digested DNA and hybridized, separately, with probes specific for the *ssp3 *or *catP*. The hybridized probe was detected using a DIG-chemiluminescence detection system utilizing CSPD^® ^[disodium 3-(4-methoxyspiro[1]-4-yl)phenyl phosphate] ready-to-use substrate (Roche) as previously described [3,4].

### CPE Western blot analysis

*C. perfringens *strains grown in DS or FTG medium were sonicated until >95% of all cells were lysed (lysis was continuously monitored by phase-contrast microscopy). After sonication, each culture lysate was analyzed for the presence of CPE by Western blot analysis using a CPE antibody as previously described [4,22].

### Determination of *D *values for *C. perfringens *spores

The heat sensitivities of *C. perfringens *spores was determined as described previously [4]. Briefly, DS medium cultures prepared and grown for 24 h as described above were heat shocked at 75°C for 20 min which killed the remaining vegetative cells and facilitated spore germination. A 0.1 ml aliquot of each heat-shocked DS medium culture was then serially diluted with sterile FTG medium and each dilution was plated onto Brain Heart Infusion (BHI) agar plates to establish the number of viable spores per milliliter of DS medium culture at the start of heating (i.e., the zero time point of the experiment).

The remainder of each heat-shocked DS medium culture was then heated at 100°C for time period ranging from 30 min to 90 min. At each time point, a 0.1-ml aliquot was withdrawn and diluted with FTG broth. The dilutions were then plated on BHI agar plates, which were incubated anaerobically at 37°C for 24 h. Colonies which developed from germinated spores that survived heating were counted to determine the number of viable colony forming unit (CFU) that were present per milliliter of each heated DS medium culture at each time point. The CFU values were then graphed to determine decimal reduction value (*D *value) (i.e., the time that a culture had to be kept at a given temperature to obtain a 90% reduction in viable cell numbers) for spores of each isolate tested.

### Assay of SASP-nucleic acid binding

The binding of *C. perfringens *SASPs to nucleic acid was assessed by measuring the ability of SASP to protect nucleic acid from nuclease digestion as previously described [20]. Briefly, SASPs was prepared from *C. perfringens *SM101 spores as described above and quantified using the Bradford protein assay kit (Bio-Rad). 3 μg of plasmid pJIR751 DNA was incubated in 25 μl of 10 mM Tris-acetate (pH 7.0) -1 mM EDTA with 3 μg of SASPs (1:1 ratio). After 1 h at 37°C, 3 μl of DNase I buffer was added, followed by 2 μl of DNase I (Farmentas). The solution was incubated a further 10 min at 37°C, and then 100 μl of 1.25% sodium dodecyl sulfate (SDS)-25 mM EDTA was added, followed by 13 μl of 5 M NaCl and 350 μl of ethanol to precipitate the DNA. The precipitated DNA was dissolved in 10 μl of water and subjected to agarose (1.5%) gel electrophoresis. The presence or absence of DNA was observed under UV illumination after staining with ethidium bromide.

### Statistical analyses

Statistical analyses were performed with student's *t *test.

## Authors' contributions

DR carried out most of the experiments, and participated in the discussions on the study design, analyses and interpretation of the data, and in the writing of the manuscript. MW carried out experiments related to isolation of SASPs from *C. perfringens*. PS participated as a consultant and hosted MW in his laboratory to learn basic techniques for isolation of SASPs. MRS, as a principal author of this manuscript, participated in the planning and designing of the experiments and writing of the manuscript.

## Figures and Tables

**Table 1:** Bacterial strains and Plasmids used in this study

Bacterial strain/Plasmid	Relevant Characteristics^a^	Sources or Reference
***C. perfringens***		
SM101	Electroporatable derivative of food poisoning type A isolate, NCTC8798, carries a chromosomal *cpe*	[28]
NCTC8239	Food poisoning type A isolate carrying a chromosomal *cpe*	[30]
FD1041	Food poisoning type A isolate carrying a chromosomal *cpe*	[30]
NCTC10239	Food poisoning type A isolate carrying a chromosomal *cpe*	[30]
C1841	Food poisoning type A isolate carrying a chromosomal *cpe*	[30]
13	Type A, laboratory strain	[18]
IH101	*spo0A *mutant derivative of SM101	[19]
DR101	*ssp3 *mutant derivative of SM101	This study
		
***Plasmids***		
pJIR 751	*C. perfringens *– *E. coli *shuttle vector ; Em^r^	[25]
pJIR 418	*C. perfringens *– *E. coli *shuttle vector ; Cm^r^, Em^r^	[31]
pMRS104	Suicidal plasmid, no origin of replication for Clostridia; Em^r^	[19]
pSM104	*cpe*-*gusA *in pJIR750; Cm^r^	[26]
pSM242	*sigK-gusA *fusion in pJIR750; Cm^r^	S.B. Melville
pMRS127	*sigK-gusA *fusion in pJIR751; Em^r^	This study
pMRS130	pJIR751 carrying promoter-less *gusA*; Em^r^	This study
pBH2	*ssp3 *and 1.9-kb each upstream and downstream region cloned into pCR^®^-XL-TOPO^®^.	This study
pSG11	*ssp1 *promoter in pCR^®^-XL-TOPO^®^	This study
pSG12	*ssp1-gusA *in pMRS127; Em^r^	This study
pDR13	*ssp2 *and 1.4-kb each upstream and downstream region cloned into pCR^®^-XL-TOPO^®^.	This study
pDR14	*Nru*I site incorporated into *ssp2 *in pDR13	This study
pDR17	*ssp3 *with its promoter in pCR^®^-XL-TOPO^®^	This study
pDR18	*ssp3 *with its promoter in pJIR751; Em^r^	This study
pSG21	*ssp2 *promoter in pCR^®^-XL-TOPO^®^	This study
pSG22	*ssp2-gusA *in pMRS127; Em^r^	This study
pDR26	*ssp2*::*catP *in pCR^®^-XL-TOPO^®^	This study
pDR27	*ssp2*::*catP *in pMRS104; Em^r^Cm^r^	This study
pSG31	*ssp3 *promoter in pCR^®^-XL-TOPO^®^	This study
pDR 31	*ssp1 *and 1.5-kb each upstream and downstream region cloned into pCR^®^-XL-TOPO^®^.	This study
pSG32	*ssp3-gusA *in pMRS127; Em^r^	This study
pMRS60	*Hpa*I site incorporated into *ssp3 *in pBH2	This study
pMRS61	*ssp3*::*catP *in pMRS60; Cm^r^Km^r^	This study
pMRS62	*ssp3*::*catP *in pMRS104; Em^r^Cm^r^	This study
pDR61	*Nru*I site incorporated into *ssp1 *in pDR31	This study
pDR62	*ssp1*::*catP *in pDR61; Cm^r^Km^r^	This study
pDR63	*ssp1*::*catP *in pMRS104; Em^r^Cm^r^	This study

a. Em^r^, Cm^r^, Km^r^: resistance to erythromycin, chloramphenicol and kanamycin, respectively.

**Table 2:** Primers used in this study

Primers name	Primer Sequence^a^	Position^b^	Gene	Use^c^
CPP 7	5' GCTTACAAATTACCAAAGCC 3'	-36 to -17	*ssp1*	PCR, Se
CPP 8	5' CAGTATTAGCGAAAGGTTTG 3'	83 to 202	*ssp1*	PCR, Se
CPP 9	5' CTCCTATAATTCCCTCTCAT 3'	-105 to -86	*ssp2*	PCR, Se
CPP10	5' GTAGACTTTAATAGGTTCAGG 3'	180 to 200	*ssp2*	PCR, Se
CPP11	5' CTGCACATCATAATATTGAAAGG 3'	-80 to -58	*ssp3*	PCR, Se
CPP 13	5' GAGGGTCCTATTGTAGGAGGATT 3'	-2010 to -1888	*ssp3*	MP, SB
CPP 16	5' ATAGCAGGAGGAGCTATTCCAC 3'	2099 to 2120	*ssp3*	MP, SB
CPP 21	5' CGGAGACCTTAGCTCAAGTTAACGCGGAAGCGTTGGTGGAG 3'	95 to 135	*ssp3*	SDM
CPP 22	5' CTCCACCAACGCTTCCGCGTTAACTTGAGCTAAGGTCTCCG 3'	95 to 135	*ssp3*	SDM
CPP 34	5' GCTATGGATCTTATGGAAGG 3'	423 to 442	*ssp3*	PCR, Se
CPP37	5' CGGCTTCTAGCACATCTTCT 3'	-1662 to -1593	*ssp2*	MP, SB
CPP38	5' TATGTGGAGCAGGAATTGCC 3'	1449 to 1469	*ssp2*	MP, SB
CPP47	5'GTACCATTCTCAGATTACAATGTCGCGAAGCGTTGGTGGAGAAATGG3'	73 to 113	*ssp2*	SDM
CPP48	5' CCATTTCTCCACCAACGCTTCGCGACATTGTAATCTGAGAATGGTAC 3'	73 to 113	*ssp2*	SDM
CPP45	5' CCAGGAAAGTATGGACAAGC	-1564 to -1544	*ssp1*	MP
CPP139	5' CCTCACCATTATCCTCTACAAG 3'	1782 to 1802	*ssp1*	MP
CPP104	5' GCTAGAGAATTAGGGGTACCTCGCGACCTAAGTTCAAGACAA 3'	60 to 116	*ssp1*	SDM
CPP105	5' TTGTCTTGAACTTAGGTCGCGAGGTACCCCTAATTCTCTAGC 3'	60 to 116	*ssp1*	SDM
CPP 57	5' GCGTCGACCTGTTTGAGCTTTTTTC 3'	-200 to -213	*ssp3 *promoter	GUS
CPP 58	5' GCTGCAGCCTGGAACTAAATGTTGT 3'	3 to 21	*ssp3 *promoter	GUS
CPP 63	5' GCGTCGACTAGGTGCAGAAGC 3'	-219 to -203	*ssp1 *promoter	GUS
CPP 64	5' GCTGCAGCTGGAACTAATGATTTTGAC 3'	3 to 21	*ssp1 *promoter	GUS
CPP 65	5' GCGTCGACGGAACTAAAGCTAAATTTGG 3'	-238 to -223	*ssp2 *promoter	GUS
CPP 66	5' GCTGCAGGCTTCTGGTACTAAATGTTGTG 3'	3 to 25	*ssp2 *promoter	GUS

^a ^Restriction sites are marked by underlining.

^b^The nucleotide position numbering begins from the first codon and refers to the relevant position within the respective *ssp *gene sequence [18].

^c^PCR, polymerase chain reaction; Se, sequencing studies; MP, construction of mutator plasmid; SB, southern blot analysis; SDM, site-directed muatgenesis. GUS, construction of plasmid for β-glucuronidase assay.
